# Donor/Recipient HLA Molecular Mismatch Scores Predict Primary Humoral and Cellular Alloimmunity in Kidney Transplantation

**DOI:** 10.3389/fimmu.2020.623276

**Published:** 2021-03-10

**Authors:** Maria Meneghini, Elena Crespo, Matthias Niemann, Alba Torija, Nuria Lloberas, Vincent Pernin, Pere Fontova, Edoardo Melilli, Alexandre Favà, Nuria Montero, Anna Manonelles, Josep Maria Cruzado, Eduard Palou, Jaume Martorell, Josep Maria Grinyó, Oriol Bestard

**Affiliations:** ^1^Kidney Transplant Unit, Nephrology Department, Bellvitge University Hospital, Barcelona, Spain; ^2^Translational Transplantation and Nephrology Laboratory, Institut d'Investigació Biomèdica de Bellvitge (IDIBELL), Barcelona, Spain; ^3^Director of Technology, PIRCHE-II AG, Berlin, Germany; ^4^Department of Nephrology, Dialysis and Transplantation, Montpellier University Hospital, Montpellier, France; ^5^Institute for Regenerative Medicine & Biotherapy (IRMB), University of Montpellier, INSERM, Montpellier, France; ^6^Laboratory of Immunology and Histocompatibility, Hospital Clinic, Barcelona, Spain

**Keywords:** alloreactive, T cell, HLA mismatch, donor-specific antibodies, kidney transplantation

## Abstract

Donor/recipient molecular human leukocyte antigen (HLA) mismatch predicts primary B-cell alloimmune activation, yet the impact on *de novo* donor-specific T-cell alloimmunity (dnDST) remains undetermined. The hypothesis of our study is that donor/recipient HLA mismatches assessed at the molecular level may also influence a higher susceptibility to the development of posttransplant primary T-cell alloimmunity. In this prospective observational study, 169 consecutive kidney transplant recipients without preformed donor-specific antibodies (DSA) and with high resolution donor/recipient HLA typing were evaluated for HLA molecular mismatch scores using different informatic algorithms [amino acid mismatch, eplet MM, and Predicted Indirectly Recognizable HLA Epitopes (PIRCHE-II)]. Primary donor-specific alloimmune activation over the first 2 years posttransplantation was assessed by means of both dnDSA and dnDST using single antigen bead (SAB) and IFN-γ ELISPOT assays, respectively. Also, the predominant alloantigen presenting pathway priming DST alloimmunity and the contribution of main alloreactive T-cell subsets were further characterized *in vitro*. Pretransplantation, 78/169 (46%) were DST+ whereas 91/169 (54%) DST−. At 2 years, 54/169 (32%) patients showed detectable DST responses: 23/54 (42%) dnDST and 31/54 (57%) persistently positive (persistDST+). 24/169 (14%) patients developed dnDSA. A strong correlation was observed between the three distinct molecular mismatch scores and they all accurately predicted dnDSA formation, in particular at the DQ locus. Likewise, HLA molecular incompatibility predicted the advent of dnDST, especially when assessed by PIRCHE-II score (OR 1.014 95% CI 1.001–1.03, p=0.04). While pretransplant DST predicted the development of posttransplant BPAR (OR 5.18, 95% CI=1.64–16.34, p=0.005) and particularly T cell mediated rejection (OR 5.33, 95% CI=1.45–19.66, p=0.012), patients developing dnDST were at significantly higher risk of subsequent dnDSA formation (HR 2.64, 95% CI=1.08–6.45, p=0.03). *In vitro* experiments showed that unlike preformed DST that is predominantly primed by CD8+ direct pathway T cells, posttransplant DST may also be activated by the indirect pathway of alloantigen presentation, and predominantly driven by CD4+ alloreactive T cells in an important proportion of patients. *De novo* donor-specific cellular alloreactivity seems to precede subsequent humoral alloimmune activation and is influenced by a poor donor/recipient HLA molecular matching.

## Introduction

Long-lasting survival of kidney transplantation is greatly challenged by both preformed and primary donor-specific humoral alloimmunity: the former preventing access to transplantation in sensitized patients, and the latter accelerating chronic rejection and premature graft loss (1, 2). Between 5 and 9% of kidney transplant recipients may develop *de novo* donor-specific antibodies (dnDSA) each year mainly against class-II human leukocyte antigens (HLA) (3, 4). This is of significant clinical relevance, being chronic antibody mediated rejection (ABMR) one of the leading causes of death-censored graft loss that may explain to some extent why even with modern immunosuppression, long term graft survival has not improved in recent years (5).

Recent data show that a major determinant of primary humoral alloimmune activation relies on poor donor/recipient HLA matching, especially in case of non-adherence or insufficient immunosuppression exposure (4, 6, 7). Notably, while clinical histocompatibility assessment is still based on alphanumeric class-I/II allele matching, novel computed algorithms have refined its evaluation by assessing the mismatch (MM) degree at a molecular level (8). The definition of the molecular differences between donor and recipient HLA molecules has been an interesting field of research developed in the last decade that led to the creation of informatic algorithms available for research purposes and whose clinical impact on outcomes has been investigated. On the one hand, the calculation of the number of highly polymorphic aminoacids composing the mismatched donor HLA molecules (amino acid MM) has been proposed and showed to predict primary humoral responses (9, 10). Similarly, the HLAMatchmaker algorithm defines the count of specific mismatched polymorphic aminoacidic-residues within 3 Ångstroms radius (eplets) exposed on the HLA molecular surface and constituting the functional epitopes against which anti-HLA antibodies are directed (11). The number of eplets that are mismatched between donor and recipients can be calculated by the HLAMatchmaker software either at each HLA locus, by class (1 or 2) or as a cumulative number or “eplet MM load”. Some but not all eplets have been “antibody verified” *in vitro* and since this process is ongoing, newer versions of the calculator are periodically released including the last updates on the eplets’ repertoire. An increasing number of eplet MM has been shown to identify kidney transplant recipients at higher risk of developing dnDSA, antibody-mediated rejection (ABMR) and worse allograft survival (12–15). Furthermore, since dnDSA can only be produced by B cells activated by cognate interactions with indirectly primed alloreactive T cells that have previously recognized donor HLA antigens (16), another HLA matching algorithm was developed to predict the number of recognizable donor-HLA-derived peptides that can be processed and presented by recipient’s HLA class-II molecules according to the physico-chemical characteristics of donor and recipient HLA molecules (PIRCHE-II). The PIRCHE-II score sums the number of these peptides and defines the risk of primary anti-donor humoral alloimmune activation through indirect pathway of antigen presentation. In clinical studies, this score has also been associated to the risk of dnDSA formation and graft loss (13, 17).

While previous clinical reports suggest that alloreactive T-cell priming precedes humoral activation, (4, 18, 19) there is no evidence yet showing the frequency of *de novo* donor-specific T-cell alloimmune activation (dnDST) after kidney transplantation and its association with donor/recipient HLA molecular matching. Hence, we here investigated the association of distinct donor/recipient HLA molecular mismatch algorithms with the risk of dnDST activation as well as its influence on subsequent dnDSA formation. While there are no readily available tests to monitor the presence of donor-specific T-cell responses in the clinical setting, we used one of the most sensitive immune assays tracking circulating frequencies of donor-reactive memory/effector T cells, the IFN-γ Enzyme-Linked ImmunoSpot (ELISPOT), which has been validated between different research consortiums (20, 21) and has shown important associations between preformed T-cell alloimmune memory and posttransplant rejection risk (22–24). On the other hand, the development of single antigen beads using solid phase assays has revolutionized the field of humoral alloimmune risk-stratification as the most reliable assay tracking anti-HLA antibodies in clinical practice (25). Therefore, to obtain a complete picture of the kinetics of posttransplant donor-specific alloimmune responses, we used these two immune assays to detect dnDST and dnDSA at different time points during the first 2 years after kidney transplantation. Finally, the role of main T-cell subsets accounting DST alloreactivity and the type of alloantigen presenting pathways priming dnDST *in vitro* were further assessed to characterize the predominant donor-antigen T-cell priming occurring after transplantation.

## Material and Methods

### Patients of the Study

**Figure 1:**
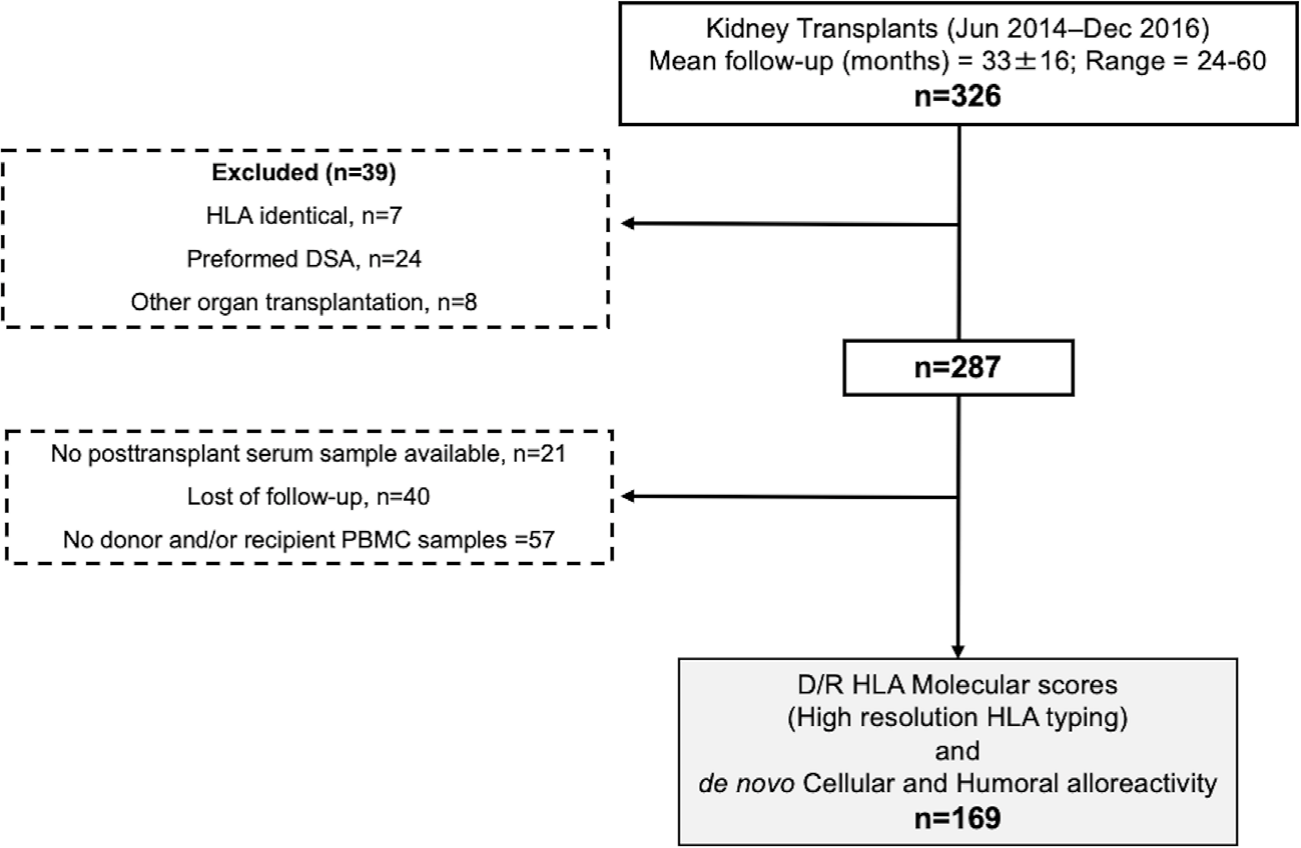
Flow chart of the study. PBMCs, peripheral blood mononucleated cells; DSA, donor specific antibody.

As illustrated in **Figure 1**, between June 2014 and December 2016 326 adult kidney transplants were performed. Out of them, multiorgan transplant recipients, ABO incompatible, and HLA identical transplant recipients, with preformed DSA, without available donor/recipient PBMCs and/or high-resolution HLA typing and those lost to follow-up were excluded from this study. Clinical data were collected prospectively during clinical follow-up. BPAR was defined according to latest BANFF classification (26). Graft loss was defined either as re-transplantation or return to chronic renal replacement therapy. Minimum patient follow-up was 2 years (mean: 33 ± 16 months, range 24–60). All patients signed informed consent to participate in the study, which had been previously approved by the local Investigator Research Board.

### HLA Typing and Donor/Recipient Mismatch Scores

#### HLA Typing

High-resolution donor and recipient HLA typing was done for both class-I (A, B, C) and class-II (DRB1, DQB1, DPB1) antigens with NGS technology. Exons 2, 3, 4 for class I and exons 2 and 3 for class II were amplified by multiplex PCR. NGS was performed on a MiSeq platform (Illumina, San Diego, California). DRB3/4/and DPA1 could not be assessed in all donor/recipient pairs because of insufficient biological material, thus HLA mismatch scores were performed at A, B, C, DRB1, DQB1 and DPB1 loci. Notably, since all recipients could be typed for DQA1, we evaluated PIRCHE score also taking into account the alloantigen presentation by recipients’ DQ(B1/A1)+DRB1 molecules.

#### Amino Acid HLA Mismatches

The HLA epitope mismatch algorithm (HLA‐EMMA) was used to assess polymorphic amino acids on mismatched donor HLA molecules as previously described (27). Both total amino acid sequences and amino acids in solvent accessible positions were assessed as a global score and at the single HLA locus or molecule. The software package is available at http://www.HLA-EMMA.com.

#### HLAMatchmaker Algorithm

The HLAMatchmaker program (Rene Duquesnoy, 2016, University of Pittsburgh Medical Center, Pittsburgh, PA HLA-ABCEpletMatchingVersion3.1 and DRDQDPEpletMatchingProgramV3.1 from http://www.epitopes.net/downloads.html) was used to calculate eplet scores as previously described (6). Total number of eplet and antibody verified eplet mismatches were calculated for all HLA molecules (eplet MM load), for each locus and for each donor HLA molecule separately.

#### Predicted Indirectly Recognizable HLA Epitopes II Algorithm

PIRCHE-II score was calculated as previously described using the latest version3.3 from https://www.pirche.org (28). Briefly, the NetMHCIIpan3.0 algorithm was used to predict the non-americ-binding cores of donor mismatched HLA-derived peptides that can bind to recipient HLA-DRB1. Relevant HLA-DRB1 binders were defined as peptides with an IC50<1,000nM for HLA-DRB1 (15).

Donor-derived HLA class-II binder peptides that differed at least one amino acid in their non-americ-binding core from recipient’s HLA sequence were counted as PIRCHE-II. Donor epitope-HLA complexes that were present multiple times in a donor/recipient couple were counted as a single PIRCHE-II. The analysis of PIRCHE-II global score enumerates all class I/II donor derived peptides, presented by recipient DRB1 molecule. The peptide counts originated from each donor’s locus and each donor molecule is also described. The analysis of DRB1 and DQ presentation of donor-derived peptides, and different IC50 cut-offs for peptide binding, were evaluated.

### Anti-HLA Antibody Determinations

Patients’ sera were tested for the presence of class-I and II anti-HLA IgG antibodies at baseline, 6 and 12 months after transplantation and annually thereafter. A single-antigen class-I and class-II flow beads-assay kit was used (LIFECODES, division of Immucor, Stanford, CA). All beads showing a normalized MFI>500 were considered positive if (MFI/MFI lowest bead)>5.

### Donor and Recipient Peripheral Blood Mononuclear Cell Samples Preparation and Evaluation of Circulating Donor-Specific T-Cell Alloreactivity

#### Donor and Recipient Peripheral Blood Mononuclear Cell Samples

Recipient and donor PBMCs from living donors or splenocytes from deceased donors were harvested and isolated by Ficoll density gradient centrifugation. Donor samples were depleted from T-Cells using either anti-CD3 (Human CD3+Cell Depletion Kit-RosetteSep Kit, STEMCELL, France) or anti-CD2 kits (EasySep1 Human-CD2 Selection Kit, STEMCELL, France), in living or deceased donors, respectively, to avoid any donor T-cell alloimmune response. All samples were frozen in liquid nitrogen at −80°C until their use.

#### Evaluation of Donor-Specific Alloreactive T-Cell Responses

The assessment of DST in peripheral blood, both prior and posttransplantation, was done using the IFN-γ Enzyme-linked Immunosorbent Spot (ELISpot) assay as previously described (21). Briefly, 3x10^5^ responder PBMC were placed in each Elispot well plate coated with primary IFN-γ antibody wells with 3x10^5^ donor cells, in triplicates. A negative control (complete medium alone: RPMI 1640, GE Healthcare Life Sciences, USA, with 10% inactivated FBS, antibiotics and L-glutamine) and a positive control (Pokeweed, AID, Autoimmun Diagnostika) were also tested in duplicates. Incubation time was 22 h at 37°C, 5% CO2. Results were expressed as frequencies of IFN-γ producing T-cells/3x10^5^ PBMCs, subtracting responses from negative donor and recipient control wells. As previously reported, a cut off of ≥25 spots/3x10^5^ PBMCs was considered positive (21, 29).

#### Analysis of T-Cell Receptor Dependent Activation-Induced T-Cell Markers

To assess the contribution of CD8 and/or CD4 T-cell subsets to the allogenic T-cell response assessed *in vitro*, 22 donor-recipient pairs with remaining available samples (pretransplant DST−, n=5; pretransplant DST+, n=10; dnDST+, n=7) were tested in a T-cell receptor (TCR)-dependent Activation-Induced T-Cell Markers (AIM) assay as previously described (30).

Cells were cultured in 96-wells round bottom plates at 3x10^5^PBMC per well either with 100μl of medium (negative control), 3x10^5^ T-cell depleted donor cells (allo-stimulation) or 50μl of phytohemagluttinin-PHA (positive control). After incubation, cells were stained with the following antibodies: CD4-FITC, CD8-APC-H7, CD134 (OX-40Antigen)-PE, CD69 (very early activation antigen)-PE-Cy7, CD137 (4-1BB)-APC, 7-AAD (BD Biosciences, San Diego, CA). Donor Cells after 22 h incubation with medium were stained with CD4-FITC, CD8-APC-H7 antibodies to test effective T-cell depletion. After 22 h incubation with T-cell depleted donor cells, we assessed by flow cytometry analysis the % of AIM+ cells defined as the % of (CD69+CD137+) cells for CD8+ T cells, and (CD134-OX40+ CD137+) for CD4+. T-cell activation results are presented by subtracting the percentage of AIM+ cells after stimulation with medium (negative control) from % of AIM+ cells after allogenic stimulation.

Flow cytometry was performed on a FACS-Canto flow cytometer and analyzed using the FACS-Diva Software (BD Biosciences, San Diego, CA).

#### *In Vitro* Assessment of Alloantigen-Presenting Pathways Priming Donor-Specific T Cells

In order to characterize the predominant alloantigen-presenting pathways of circulating DST *in vitro*, a subset of DST+ patients with available cell samples, either prior and/or after transplantation were functionally re-evaluated (preDST+, n=9; dnDST+, n=9; persistDST+, n=9). For these experiments we modified the functional immune assay by evaluating in the same patient DST responses with the following conditions: 1) using total recipient PBMC as responder cells co-cultured with T-cell depleted donor stimulating cells and, 2) using recipient T-Cells only after being selectively isolated as responder cells co-cultured with donor stimulating cells. In the first assay, both directly and indirectly primed DST frequencies are detected, since recipient PBMCs include T cells (CD3+), B cells (CD19+), monocytes (CD14+) and dendritic cells (HLADR+CD14− CD3− CD19−CD56−) (**Supplementary Figure 1**), whereas in the second experiment only T cells are present as responders thus, DST frequencies primed by the direct pathway (DP) of antigen presentation may be only detected. For these later experiments, a positive selection of recipient CD3+ T-Cells was done (Human T Cell Enrichment Kit-RosetteSep Kit, STEMCELL, France). Importantly, the same number of CD3+ T-Cells present in the all PBMCs sample was seeded in each well when analyzing the DST with enriched responder T-Cells, to avoid any additional response due to higher presence of responder T-Cells. Therefore, to assess the relative role of indirectly primed (IP) DST cells in the *in vitro* assays, the total number of IFN-γ spots observed in the DP experiment was subtracted from those observed in the same patient when using all PBMCs as responder cells. PBMC subsets were stained with combinations of the following fluorochrome conjugated antibodies: CD3-APC-H7, CD19-FITC, CD14-PECy7, CD56-PE, HLADR-APC (BD Biosciences, San Diego, CA).

### Statistical Analysis

All continuous data are presented as mean ± SD or median and interquartile-range. Different groups were compared using X^2^ test for categorical variables and student t-test for normally distributed data, and non-parametric Kruskal-Wallis or Mann-Whitney U test for non-normally distributed variables. Bivariate correlation analyses were performed by Pearson or Spearman test (non-parametric variables). Univariate and multivariate logistic regression analyses were used to determine the variables associated with the risk of developing BPAR and dnDST. The time-dependent association of the variables assessed on graft survival and dnDSA development was studied with Cox proportional hazard, Kaplan–Meier plots, and log-rank test. The statistical significance level was defined as 2-tailed p<0.05. Statistical analyses were performed with IBM SPSS Statistics, version 26 (Armonk, NY) and GraphPad Prism version6.0 (GraphPad Software, La Jolla, CA).

## Results

### Patients of the Study and Main Clinical Outcomes

As illustrated in **Figure 1**, 169 consecutives non HLA-identical, single, adult transplant recipients at Bellvitge University Hospital (Barcelona, Spain) without preformed DSA and in whom both donor and recipient HLA typing was characterized using high resolution Next Generation Sequencing (NGS) technology and peripheral blood mononuclear cells (PBMC) to monitor DST were obtained both prior and at different time points after transplantation were evaluated in this study.

As shown in **Table 1**, the patients included in the study were representative of the total kidney transplant patients performed during the study timeline, as there were no differences regarding main demographic, immunological, and clinical outcomes. Most patients of the study were male, Caucasic transplant recipients receiving a deceased donor kidney. Induction immunosuppression was mainly based on basiliximab induction with tacrolimus-based maintenance triple therapy.

**Table 1:** Main baseline and clinical outcomes of the study population and comparison with patients not included in the study.

Main baseline characteristics	All patients (n=169)	Not studied patients (n=118)	p
Recipient age (years)	52 ± 14	52 ± 14	0.83
Recipient gender (male)	110 (65)	30 (25)	0.09
Race (Caucasic)	158 (94)	113 (96)	0.41
Cause of end stage disease *Vascular* *Diabetes* *Glomerular* *Polycystic kidney disease* *Interstitial disease* *Others/unknown*	20 (12) 8 (5) 48 (28) 23 (14) 24 (14) 46 (27)	21 (18) 14 (12) 30 (25) 16 (14) 11 (9) 26 (22)	0.12
Time on dialysis (months)	25 ± 34	21 ± 25	0.23
Transplant type (deceased)	115 (68)	88 (75)	0.23
Donor age (years)	55 ± 15	54 ± 12	0.86
Transplant number (1)	152 (90)	106 (90)	0.98
Cold ischemia time (hours)	12.8 ± 9.5	11 ± 9	0.18
Pre-transplant anti-HLA			
(non DSA) antibodies			
Class I	14 (8)	10 (8.5)	0.34
Class II	17 (10)	12 (10.2)	0.30
cPRA (maximum)	2.8 ± 6.6	2.6 ± 5.9	0.88
Main immunosuppression			
- Induction	32 (19)/126 (74)/11 (6)	30 (25)/85 (72)/3 (2)	0.14
(rATG/basiliximab/none)			
- Maintenance therapy (CNI, tacrolimus)	150 (89)	116 (98)	0.06
- Steroid withdrawal before 6 months (yes)	50 (30)	38 (34)	0.41
**Main clinical outcomes**			
DGF	46 (27)	36 (31)	0.54
BPAR *TCMR/ABMR*	19 (11) 15/4	17 (14) 16/1	0.43 0.21/0.33
Patients developing *de novo* DSA	24 (14)	20	0.49
HLA class I	6	3	
HLA class II	19	18	
HLA class I and II	1	1	
Death-censored graft loss	9 (5)	10 (9)	0.32
Patient death	11 (6)	4 (4)	0.24

Data are mean (standard deviation, SD) or n (%).

cPRA, calculated panel of reactive antibodies; rATG, rabbit anti thymoglobulin; CNI, calcineurin inhibitor; BPAR, biopsy-proven acute rejection; TCMR, T cell mediated rejection; ABMR, antibody-mediated rejection; DSA, donor-specific antibodies.

Forty-six (27%) patients developed delayed graft function (DGF) and 19 (11%) biopsy-proven acute rejection (BPAR) (79% TCMR, 21% ABMR). 24/169 (14%) patients developed dnDSA: 6 (25%) class-I only (2 against A, 3 anti-B and 1 anti-C), 19 (80%) class-II only (anti-DR n=1, 5%; anti-DQ n=17, 89%, anti-DP n=1, 5%), and 1(5%) patient against both class-I and II. Five (21%) patients developed dnDSA against both donor DQ molecules, thus the majority of dnDSA were directed against DQ antigens (22/30, 73%). Mean time until first dnDSA detection was 24± 20 months (range 6–60). Mean dnDSA mean fluorescence intensity (MFI) was 8,685 (range 1,152–20,338).

Death-censored graft loss occurred in 9 (6%) patients, being main causes BPAR (5, 55%), interstitial fibrosis/tubular atrophy (2, 22%), primary glomerulonephritis recurrence (2, 22%). Eleven (6%) patients died with a functioning graft because of malignancies (5, 45%), infections (3, 27%), and cardiovascular events (3, 27%).

A detailed description of the different HLA mismatch (MM) scores of the study population is depicted in **Supplementary Table 1**. Despite the strong positive correlation between the three molecular MM algorithms, a single number of HLA allelic mismatch could correspond to a wide range of molecular MM at the individual patient level (**Supplementary Figure 2**).

No direct association was observed between BPAR and the HLA allelic, amino acid, and eplet MM scores (OR 1.08, 95% CI 0.84–1.38, p=0.54 allelic; OR 1.01, 95% CI 0.99–1.04, p=0.33 global amino acidic and OR 1.02, 95% CI 0.98–1.07, p=0.34 eplet MM), but for global PIRCHE-II score (OR 1.012, 95% CI 1.001–1.023, p=0.038). Patients developing ABMR during follow-up showed a trend towards higher amino acid MM (88±5 *vs.* 63±22, p=0.08); global PIRCHE-II (117±48 *vs.* 80±38, p=0.06) and higher eplet MM load (41.5±7 *vs.* 32±11, p=0.07). There was no effect of molecular MM scores on graft function progression, death-censored graft survival, and patient death (data not shown).

### Donor/Recipient HLA Molecular Mismatch Scores Predict Primary Humoral Alloimmunity

**Figure 2:**
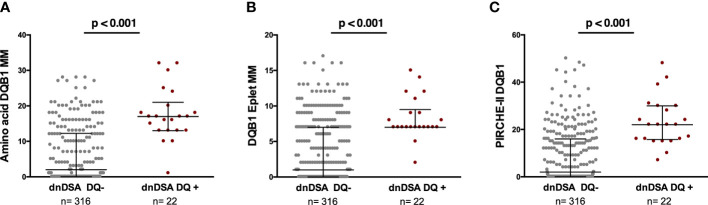
Association between amino acid MM, eplet MM load, and PIRCHE-II score for each donor DQ molecule and the respective dnDSA formation. Each dot illustrates the single molecular MM score of each donor DQ molecule against which the patients developed or not dnDSA. **(A)** Amino acid MM 6.5 ± 7.9 vs 17.6 ± 7.4, p<0.001. Solvent accessible 4.82 ± 6.18 vs 12.87 ± 6.29, p<0.001. **(B)** Eplet MM load 3.56 ± 4.33 vs 8.27 ± 2.95, p<0.001. Antibody-verified 1.31 ± 1.87 vs 2.82 ± 1.26, <0.001. **(C)** PIRCHE-II 8.98 ± 11.54 vs 23.2 ± 10.84, p<0.001. MM, mismatch; dnDSA, *de novo* donor-specific antibody.

As shown in **Figure 2**, significantly higher MM scores of each molecular algorithm against the individual mismatched donor DQ molecule was observed for the respective anti-DQ dnDSA. No association was observed at the allelic MM level (data not shown). A similar association was observed when donor DQ peptides presented by both recipient DRB1 and DQ were assessed (35.19±29 *vs.* 17.70±23, p=0.0002, in dnDSA+ *vs.* dnDSA−, respectively). We did not study the impact of the different molecular algorithms in the two solely anti-DP and anti-DR dnDSA. A positive correlation with anti DQ dnDSA MFI was observed for DQB1 amino acid MM (r=0.57, p=0.02; solvent accessible r=0.60, p=0.013), DQB1 eplet MM (r=0.44, p=0.03), and DQB1 PIRCHE-II score (r=0.36, p=0.08).

### Donor/Recipient HLA Molecular Mismatch Scores and Primary T-Cell Alloimmunity

#### Pretransplant DST Does Not Correlate With Donor/Recipient HLA Molecular MM Scores

Despite the absence of preformed DSA, 78/169 (46%) showed high frequencies of pretransplant DST (preDST+), whereas 91 (54%) did not (preDST−). No association was found between preDST+ and main clinical, demographic characteristics nor with different HLA molecular MM scores (**Supplementary Table 2**). Nonetheless, preDST+ patients showed higher risk of BPAR (OR 5.18, 95% CI=1.64–16.34, p=0.005), mostly TCMR (OR 5.33, 95% CI=1.45–19.66, p=0.012) (**Supplementary Figure 3**), whereas it was not associated with dnDSA nor death-censored graft survival. Multivariate logistic regression analysis showed that while PIRCHE-II and tacrolimus CV (OR 1.02, 95% CI 1–1.04, p=0.047) where associated to BPAR, only preDST+, induction therapy with rATG and DGF were independent correlates of BPAR (preDST+ OR 8.46, 95% CI 1.7–41.8, p=0.009; rATG induction OR 0.13, 95% CI 0.14–1.3, p=0.08; DGF OR 3.9, 95% CI 1.2–13.1, p=0.03).

#### PIRCHE-II Score Identifies Patients at Risk of Primary Donor-Specific T-Cell Alloreactivity

**Figure 3:**
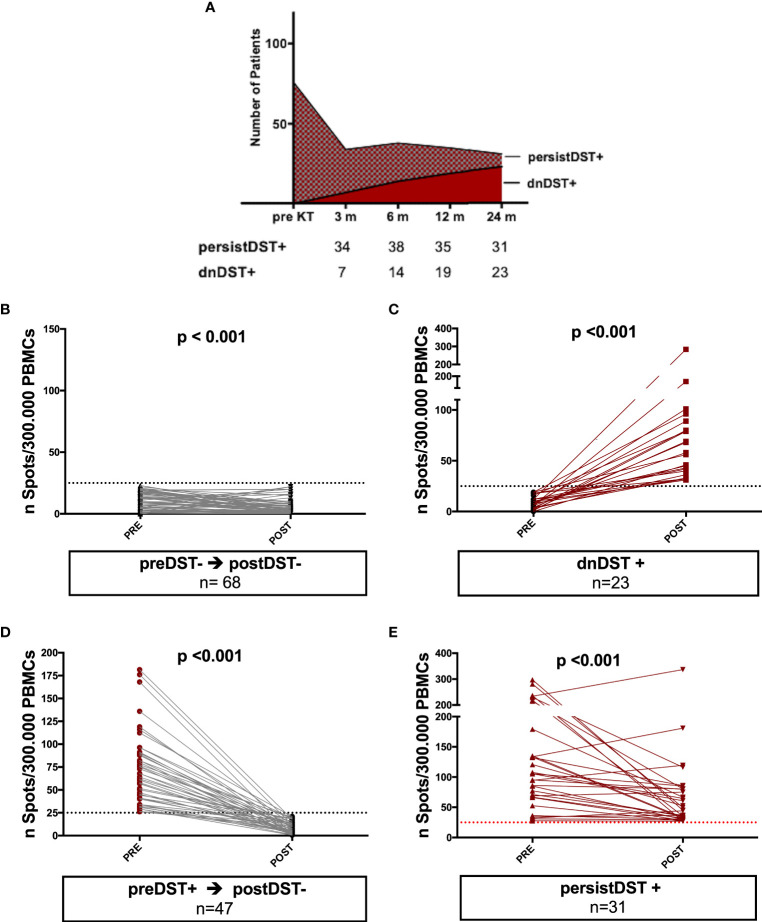
Kinetics of posttransplant *de novo* DST and changes of mean donor-reactive IFN-γ T-cell frequencies between pre and posttransplantation in different groups of patients. **(A)** At month 3, 6, 12, and 24 months 7, 7, 5, and 4 patients developed dnDST, and 34, 38, 35 and 31 were PersistDST+ respectively. **(B–E)** All preDST− remaining DST− and preDST+ becoming postDST− showed significantly lower T-cell frequencies posttransplantation. PersistDST+ although remaining positive, showed weaker responses. Only dnDST+ patients showed a significant increase of spots number. **(B)** preDST− and postDST−: preDST mean 9.76 ± 7.2 IFN-γ spots/300.000 PBMC; postDST mean 5.28± 6.27 IFN-γ spots/300.000 PBMC **(C)** dnDST: postDST mean 70.7±55.9 spots/300.000PBMCs. **(D)** preDST+ and postDST−: preDST+ mean 67.04 ± 35.9 IFN-γ spots/300.000PBMC; postDST: mean 8.76±6.35 IFN-γ spots/300.000 PBMC **(E)** persistDST+: postDST mean 66.56±61.04 IFN-γ spots/300.000 PBMC. DST, donor specific T cell alloreactivity; preDST, pretransplant donor-specific T-cell alloimmune response; postDST, posttransplant donor specific T-cell alloreactivity; dnDST, *de novo* donor specific T-cell alloreactivity; persistDST, persistent donor specific T-cell alloreactivity.

After transplantation, 54/169 (32%) patients showed DST responses at some timepoint (postDST+), being 23 (42%) dnDST and 31 (57%) persistently positive (persistDST+), whereas 115/169 (68%) were postDST− (68 preDST− and 47 preDST+) (**Figure 3A**). Changes of mean donor-reactive IFN-γ T-cell frequencies between pre and posttransplantation are depicted in **Figures 3B–E**.

**Figure 4:**
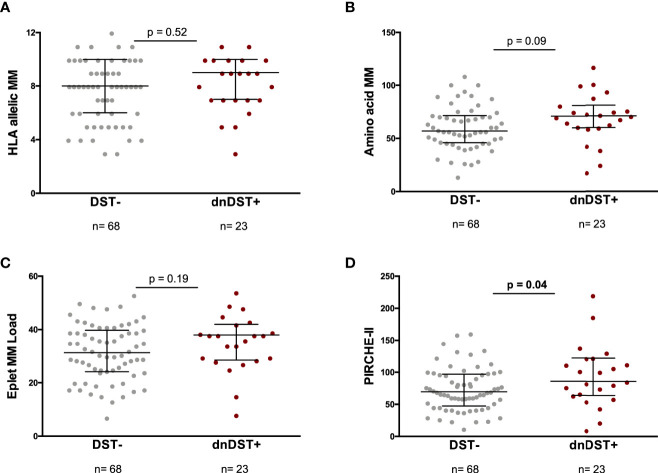
HLA allelic MM, amino acid MM, eplet MM load, global PIRCHE-II and *de novo* DST. Comparisons between HLA MM scores and dnDST- or dnDST+ patients. **(A)** HLA allelic MM 7.66 ± 2.3 vs 8.05 ± 2.2, p=0.52. **(B)** Amino acid MM 60.52 ± 20.6 vs 69.47 ± 25.8, p=0.09. **(C)** eplet MM load 31.62 ± 10.5 vs 35 ± 10.5, p=0.19. **(D)** PIRCHE-II 74.1 ± 33.2 vs 94.8 ± 48.1, p=0.04. MM, Mismatch; DST, donor specific.

While none of the different HLA MM scores associated with postDST+ (persistDST+ and/or dnDST+) (data not shown), a significantly higher global PIRCHE-II score was observed among dnDST+ than within postDST− patients (**Figure 4**). When analyzing the single HLA loci, dnDST patients showed significantly higher solvent-accessible DRB1 amino acid MM, not-Ab-verified (Abv) DRB1 eplet MM, PIRCHE-II DRB1, and PIRCHE-II DQB1 (DRB1 amino acid MM 11.17 ± 6.2 *vs.* 8.18 ± 6.4, p=0.05, not-Abv DRB1 Eplet 6.3±3.05 *vs.* 4.79±3.5, p=0.06 PIRCHE-II DRB1 15.5 ± 11.9 *vs.* 9.44 ± 8.4, p=0.03, PIRCHE-II DQB1 22.65 ± 15.7 *vs.* 16.29 ± 12.5 p=0.05). When assessing the PIRCHE-II score presented by DRB1+DQ molecules, similar results were observed, being the count of DRB1 donor-derived peptides similarly associated to dnDST activation (29.95±24.2 *vs.* 20.15±17.8, p=0.04). However, the difference in global PIRCHE-II score presented by both DRB1+ DQ molecules was not statistically different (199.4±132 in dnDST+ *vs.* 175.1±91 in dnDST−, p=0.4). The relationship between PIRCHE-II and dnDST for different peptide affinity thresholds (IC50: 0–50, 0–125, and 125–1,000), revealed that PIRCHE-II was significantly associated to dnDST especially at less stringent IC50 intervals (**Supplementary Figure 4**). Donor-specific T-cell frequencies did not correlate with amino acid MM (r=0.17, p=0.14) nor Eplet MM load (r=0.1, p=0.34), whereas showed a weak but positive linear correlation with the global PIRCHE-II score (r=0.24, p=0.025).

In the univariate and multivariate logistic regression analysis for the prediction of dnDST, high global PIRCHE-II score and delayed graft function were independent correlates (**Table 2**). Conversely, persistDST+ was only predicted by absence of T-cell depletion (OR 0.09, 95% CI 0.01–0.62, p=0.01) and high pre-transplant IFN-γ ELISpot frequencies (OR 1.02, 95% CI 1.009–1.03, p=0.001).

**Table 2:** Univariate and multivariate logistic regression for the risk of *de novo* donor-specific T-cell (dnDST).

Variable	Univariate analysis			Multivariate analysis		
	OR	95% CI	p	OR	95% CI	p
Recipient age (y)	1.02	0.99–1.06	0.21			
Donor age (y)	1.02	0.98–1.05	0.42			
Recipient gender (m)	0.96	0.35–2.59	0.93			
Donor gender (m)	1.52	0.55–4.21	0.42			
Transplant number (>1)	1.02	0.19–5.42	0.98			
Race (Caucasic)	0.48	0.08–3.1	0.44			
**Type of donor (deceased**	**4.96**	**1.34–18.3**	**0.02**	1.04	0.05–20.5	0.99
**Cold ischaemia time (hours)**	**1.08**	**1.02–1.15**	**0.01**	1.05	0.91–1.21	0.51
**DGF**	**4.67**	**1.64–13.24**	**0.004**	**4.11**	**1.18–14.3**	**0.03**
Type of induction IS (no rATG)	1.36	0.49–3.76	0.55			
Steroid withdrawal	1.40	0.45–4.29	0.56			
Type of maintenance IS (CNI)	0.85	0.26–3.07	0.85			
Tacrolimus CV%	1.004	0.98–1.03	0.76			
Previous BPAR	3.14	0.42–23.70	0.27			
HLA allelic MM	1.08	0.86–1.36	0.52			
Amino acid MM	1.02	0.99–1.04	0.13			
Eplet MM load	1.03	0.98–1.08	0.18			
**Global PIRCHE-II**	**1.014**	**1.001–1.03**	**0.03**	**1.015**	**1.001–1.03**	**0.04**

rATG, rabbit anti thymoglobulin; DGF, delayed graft function; CNI, calcineurin inhibitor; CV, coefficient of variation (CV = σ/μ × 100); BPAR, biopsy-proven acute rejection; HLA, human leukocyte antigens; MM, mismatches; IS, immunosuppression; DST, donor-specific T-cell alloimmunity. In bold are statistically significant variables.

#### *De Novo* DST Predicts Subsequent Development of dnDSA

**Figure 5:**
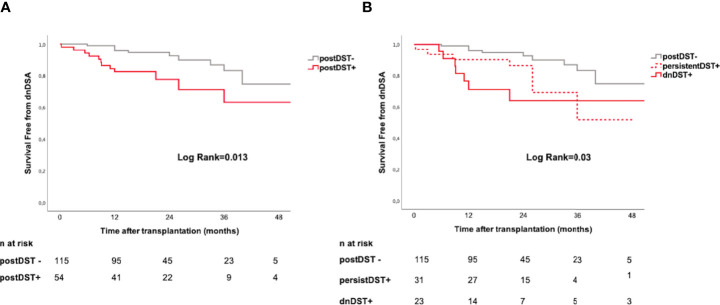
Posttransplant donor-specific T-cell alloreactivity and *de novo* DSA formation. Kaplan–Meier curves illustrating the cumulative incidence of dnDSA stratified according to: **(A)** postDST− *vs.* postDST+ **(B)** postDST+ further stratified in dnDST or persistDST. postDST− *vs.* persistDST+= log rank 0.07; postDST− *vs.* dnDST+ log rank=0.01; persistDST+ *vs.* dnDST+ log rank=0.36. dnDSA, *de novo* donor-specific antibody; DST, donor specific T-cell alloreactivity; postDST, post-transplant donor specific T-cell alloreactivity; dnDST, *de novo* donor specific T-cell alloreactivity; persistDST, persistent donor specific T-cell alloreactivity.

While postDST+ showed a higher risk of subsequent dnDSA formation (HR 2.66, 95% CI=1.19–5.95, p=0.017), when stratifying postDST in either persistent or *de novo*, dnDST displayed a stronger risk of dnDSA than persistDST (HR 2.64, 95% CI=1.08–6.44, p=0.03 and HR 1.62, 95% CI=0.63–4.13, p=0.31, respectively). Kaplan-Meier dnDSA-free survival curves illustrate the cumulative dnDSA rates among different postDST groups (**Figure 5**).

In addition, transplant patients with both dnDST+ and dnDSA+ showed significantly higher PIRCHE-II global score as compared to patients with either dnDST or dnDSA or those without dnDSA nor dnDST (101±49 *vs.* 78.9±38, p=0.04). No differences were observed with any of the other HLA molecular MM algorithms at this level.

While we did not observe any correlation between posttransplant IFN-γ ELISpot frequencies and MFI of dnDSA (Rho −0.7, p=0.75), a weak but statistically significant inverse correlation with 12 and 24-month graft function was observed (eGFR 12months r=−0.25, p=0.01; eGFR 24 months r=−0.20, p=0.01).

### Higher Involvement of CD4+ T Cells in *De Novo* T-Cell Alloreactivity as Compared to Pretransplantation

The contribution of CD8+ and CD4+ T cells to donor-reactive T-cell responses were investigated using the TCR dependent activation-induced cell marker (AIM) assay in a subset of patients. CD4+ and CD8+ AIM+ T cells varied among different DST groups, which were detected both within preDST+ and dnDST+ patients (**Supplementary Figure 5**).

**Figure 6:**
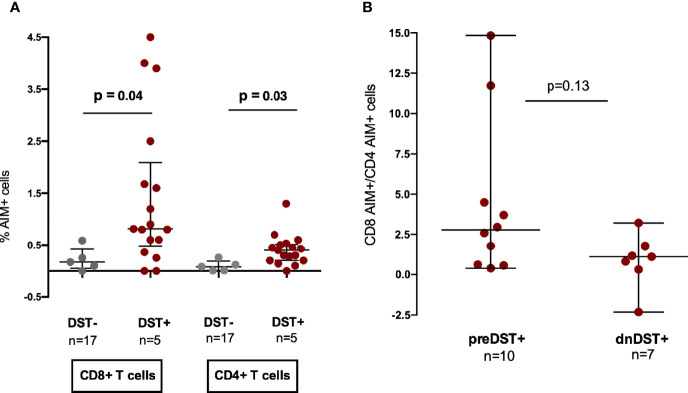
Contribution of CD8 and CD4 T cell subsets to pre- and posttransplant donor-specific alloreactivity assessed by T-cell receptor dependent activation-induced cell markers (AIM) by flow cytometry analysis. **(A)** Comparison of % of CD8+ AIM + T cells (CD69+CD137+) and AIM+ (OX40+CD137+) CD4+ T cells after allogenic (donor-specific stimulation) in DST− or DST+ patients. CD8+ AIM+: median 0.17% (0.05–0.42) vs 0.81% (0.48–2.09), p=0.041; CD4+AIM+: median 0.08% (0.008–0.19) vs 0.40% (0.2–0.56), p=0.029 in non alloreactive versus alloreactive patients, respectively. **(B)** CD8+/CD4+ AIM+ T-cell ratio in preDST+ and dnDST+ samples, respectively. Median 2.77 (0.6–6.3) in preDST+ *vs.* 1.13 (−2.3–1.79) in dnDST+, p=0.13. AIM, T-cell receptor dependent activation-induced cell markers; DST, donor specific T-cell alloreactivity; preDST, pretransplant donor specific T-cell alloreactivity; dnDST, *de novo* donor specific T-cell alloreactivity.

The percentages of CD8+ and CD4+ AIM+ T cells, were significantly higher among DST+ as compare to DST− independently of the time of the IFN-γ ELISpot test assessment, either before or after transplant (**Figure 6A**), confirming that the two assays are concordant detecting the same donor-reactive T cells. Notably, when we stratified for time of assessment, *dn*DST+ showed a numerically lower CD8+/CD4+ AIM+ T-cell ratio than preDST+ suggesting an increased contribution of CD4+ alloreactive T cells after transplantation among dnDST+ patients (**Figure 6B**).

### Contribution of Distinct Alloantigen Presentation Pathways Priming Posttransplant Donor-Specific T-Cell Alloreactivity

In order to characterize the contribution of the two main alloantigen presenting pathways, both direct (DP) and indirect (IP), priming circulating donor-reactive T cells, we functionally characterized them *in vitro*. When using whole recipient PBMC, different cell subsets other than T cells such as B cells, monocytes and dendritic cells were present, whereas only T cells were detectable when recipient PBMC were enriched for T cells (**Supplementary Figure 1**).

**Figure 7:**
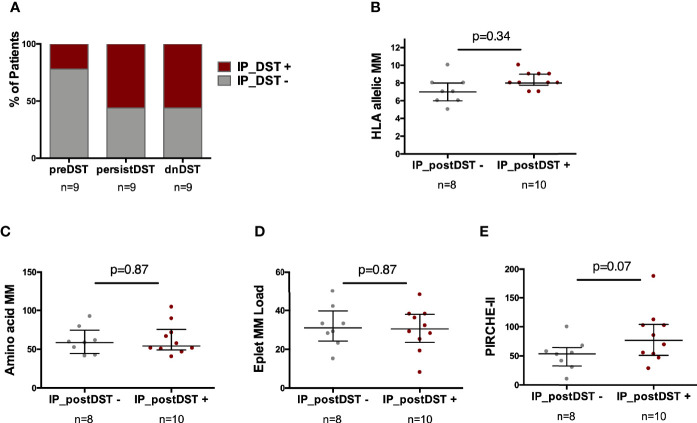
Predominance of the type of alloantigen presenting pathway priming DST according to the time of DST assessment and association with distinct HLA MM scores. **(A)** Proportion of patients showing any degree of IP_ DST+ according to timing of DST assessment (preDST n=2/9, 22%; persistDST n=5/9, 56%; or dnDST n=5/9, 56%). **(B)** Distribution of HLA allelic MM between IP_postDST− (n=8) and IP_postDST+ patients (n=10): 7.12 ± 1.5 *vs.* 8.3 ± 0.95, p=0.34 **(C)** Amino acidic MM 60.5± 17.5 *vs.* 62.5 ± 20.4, p=0.87 **(D)** Eplet MM load 31.6 ± 10.8 *vs.* 30.1 ± 11.2, p=0.87 **(E)** PIRCHE-II 51.5 ± 26 *vs.* 83.9 ± 45, p=0.07. MM, mismatch; DST, donor specific T-cell alloreactivity; preDST, pretransplant DST; postDST, posttransplant donor specific T-cell alloreactivity; dnDST, *de novo* donor specific T-cell alloreactivity; IP DST, indirect pathway donor specific T-cell alloreactivity (with recipient APCs).

While most circulating preDST+ responses [7/9, (78%)] were driven by donor-reactive T cells primed by the DP, an important proportion of patients with postDST+ responses, either dnDST+ or persistDST+, were also primed by the IP (5/9, 55% in both groups) (**Figure 7A**). While no differences were found at the HLA allelic, aminoacidic and eplet MM scores, patients with IP_postDST+ (either dnDST+ or persistDST+) showed a trend toward higher PIRCHE-II scores than those with only DP_DST+ (**Figures 7B–E** and **Supplementary Table 3**).

## Discussion

Primary humoral alloimmune activation through dnDSA production is a well-characterized deleterious factor inducing chronic ABMR and accelerated graft loss (1, 2) and recent reports have recently shown that it may be predicted by quantifying the donor/recipient HLA MM at the molecular level (6, 31). However, for B-cell activation in absence of preformed immune memory, cognate T-cell help is required thus, previous *de novo* T-cell alloimmune priming (dnDST) against donor antigens might also occur, subsequently driving anti-donor humoral immune activation.

In our study, we first confirm that HLA matching at the molecular level using distinct algorithms outperforms allelic MM assessment predicting primary humoral alloimmunity by means of dnDSA formation. Furthermore, we report that a relevant number of kidney transplant recipients develop dnDST after transplantation, which ultimately predicts the advent of dnDSA. Notably, unlike pretransplant DST, an important proportion of posttransplant DST patients, either those with persistent or *de novo* DST, display high frequencies of donor-reactive CD4+ T cells primed by the indirect antigen presentation pathway, which contributes to their global DST response. Most interestingly, and similarly to dnDSA, our data suggest that patients at risk of dnDST seem to also show a poor donor/recipient HLA molecular matching, and in particular, at the Predicted indirectly Recognizable HLA Epitopes II (PIRCHE-II) score level, emphasize the contribution of the indirect antigen presenting pathway driving DST development. These data highlight a continuous increased risk of dnDST and dnDSA for each individual predicted peptide presented by recipient APC through indirect presentation. This is, to our knowledge, the first report showing the impact of HLA molecular incompatibility on the development of primary adaptive alloimmunity, not only at the humoral but also at the cellular level in solid organ transplantation.

In order to track the presence of donor-reactive T-cell responses, we used the IFN-γ donor-specific T-cell ELISpot, a sensitive and reproducible immune-assay tracking circulating donor-reactive IFN-γ-producing memory/effector T cells (21, 32). Most studies using this test have focused on the pretransplant setting and have shown its capacity identifying transplant candidates at higher risk of BPAR, regardless preformed donor-specific humoral immune sensitization (22–24, 29). Here, while we confirm this observation, pretransplant DST was not associated with any HLA MM score thus, strongly suggesting that its presence may arise from either antigen cross-reactivity amid heterologous immunity or prior transient alloantigen recognition triggering a low immune sensitization state, predominantly at the T-cell compartment. Notably, it has recently been reported the impact of HLA class-II mismatching predicting not only the advent of dnDSA and ABMR but also TCMR (14, 33, 34). In this regard, our findings support a mechanistic explanation of incompatibility at the DR and DQ molecules being especially associated to the risk of *de novo* T-cell activation. Although intuitively, a specific threshold would be of high relevance to help stratifying patients into high or low risk for either dnDSA or dnDST, from the biological point of view these thresholds might not represent the potential impact for alloimmune activation. Indeed, despite the strong correlation between the load of molecular MM and risk of *de novo* alloimmunity, even a small amount of mismatched antigens may be sufficient to activate an immune response, thus application of specific cut-offs may be misleading in clinical practice (34, 35).

Another important observation of our study is that up to 50% of transplant recipients with preDST maintained a strong DST response after kidney transplantation, which seems to be mainly influenced by pretransplant anti-donor T-cell frequencies and the absence of T-cell depletion induction therapy. Interestingly, a strong association was observed between postDST and subsequent dnDSA formation, particularly among dnDST patients. While we cannot confirm whether patients with persistent DST show the same pretransplant donor-reactive T-cell clones after transplantation, we observed that an important proportion of them did also display DST primed by the IP, similarly to patients with dnDST thus, suggesting that DST responses among persistDST may have also been developed *de novo*. Interestingly, dnDST was also influenced by the development of delayed graft function, which could possibly be explained by an inflamed milieu with increased class II HLA antigen expression on graft cells ultimately driving T-cell alloantigen recognition. The higher presence of alloreactive CD4+ T cells in dnDST+ samples as compared to pretransplantation does also support that posttransplant anti-donor alloreactivity is driven, at least also in part, by the IP of antigen presentation. While the presence of the IP after transplantation has been widely described (16, 36, 37), a body of evidence has also shown the potential relevance of a semi-direct or third pathway of antigen presentation (38–40). In this line, we also found circulating postDST responses primed by the DP when assessed *in vitro*, most likely representing the presence of such semidirect pathway of antigen presentation *in vivo*.

Our study has some limitations. The retrospective design may hamper achieving robust conclusions. Nonetheless, the use of high-resolution HLA typing and the significant associations observed together with the concomitant mechanistic *in vitro* experiments performed, counterbalance this drawback. Also, both DPA and DRB3/4/5 typing could not be assessed, leaving undetermined the impact of molecular MM at those loci on dnDST generation as well as their peptide presenting role. Nevertheless, the accurate prediction of dnDST by donor-derived DRB1 peptides and also when evaluating DQ presentation strengthens the consistency of our findings. Notably, dnDST was accurately predicted by donor-derived DRB1 peptides but not by the global peptide burden if DQ presentation is evaluated. The expression of DQ molecules in recipient APC or different activation capacity of CD4+ T cells according to distinct HLA class-II molecules may explain this observation.

In conclusion, we here show the impact of novel HLA molecular matching scores, also influencing a higher risk of primary anti-donor cellular alloimmune activation after kidney transplantation, which seems to precede the subsequent development of *de novo* humoral alloreactivity. Importantly, the value of implementing these novel donor/recipient HLA matching scores in kidney transplantation to refine current immune-risk stratification needs to be further explored in larger studies.

## Data Availability Statement

The raw data supporting the conclusions of this article will be made available by the authors, without undue reservation.

## Ethics Statement

The studies involving human participants were reviewed and approved by Investigator Research Board Bellvitge University Hospital. The patients/participants provided their written informed consent to participate in this study.

## Author Contributions

OB: conceptualization of the study and supervision. OB, JG, MM, EM, AF, NM, AM, JC: clinical patients’ follow-up. MM, EC, AT, NL, VP, PF: performed the experiments. MM, EC, OB: collected and organized the data and performed the statistical analysis. MN, EP, JM: supervised the HLA molecular MM analysis. MM and OB wrote the first draft of the manuscript. OB and JG reviewed the manuscript. All authors contributed to the article and approved the submitted version.

## Funding

This work was supported by the Instituto de Salud Carlos III (ISCIII) (grant numbers ICI14/00242 and PI16/01321, PI19/01710) (co-funded by European Regional Development Fund, ERDF, a way to build Europe) ant the Biomarker-Driven Immunosuppression Minimization (BIO-DRIM) Consortium (EU FP7-health, grant agreement number 305147; FP7/2007-2013). Also, this work was partly supported by the SLT002/16/00183 grant, from the Department of Health of the Generalitat de Catalunya by the call “Acció instrumental de programes de recerca orientats en l’àmbit de la recerca i la innovació en salut.” The authors thank the Research Centers of Catalonia (CERCA) Programme/Generalitat de Catalunya for institutional support. OB was awarded with an intensification grant from the “Instituto de Salud Carlos III” [INT19/00051]. MM received a fellowship grant from ESOT (European Society for Organ Transplantation).

## Conflict of Interest

MN is an employee of PIRCHE AG that runs the PIRCHE web-portal.

The remaining authors declare that the research was conducted in the absence of any commercial or financial relationships that could be construed as a potential conflict of interest.
