# Supplementary material for: Donor/Recipient HLA Molecular Mismatch Scores Predict Primary Humoral and Cellular Alloimmunity in Kidney Transplantation
Source: Front Immunol. 2021 Mar 10;11:623276. doi: 10.3389/fimmu.2020.623276 (PMC7988214; doi:10.3389/fimmu.2020.623276)
Supplement: Supplementary file 1 [file DataSheet_1.pdf]

Supplementary Material

Supplementary Figures

Supplementary figure 1.

1. Peripheral blood mononuclear cell populations before and after T cell enrichment. Representative FACS plot gating strategy used to characterize the PBMC population (**1a**) and after T cell Enrichment (**1b**). **1c.** Variation in % of CD3+CD14-CD19- cells: 65.68±9.0 vs 87.9±5.7 in all PBMCs and T cell Enrichment samples respectively, p<0.001. Results from 25 independent experiments. **1d.** Variation in % of the other cells assessed: CD14+: 9.44±3.7 vs 2.5±1.9, p=0.002; CD19+CD14-: 2.08±0.84 vs 0.96±0.3, p=0.04; CD56+ CD14- CD3-CD19-: 8.68±6.8 vs 3.85±3.7, p=0.03; HLADR+CD14-CD19-CD3-CD56-: 1.650.5 vs 0.87±0.3, p=0.03 in All PBMCs and T cell

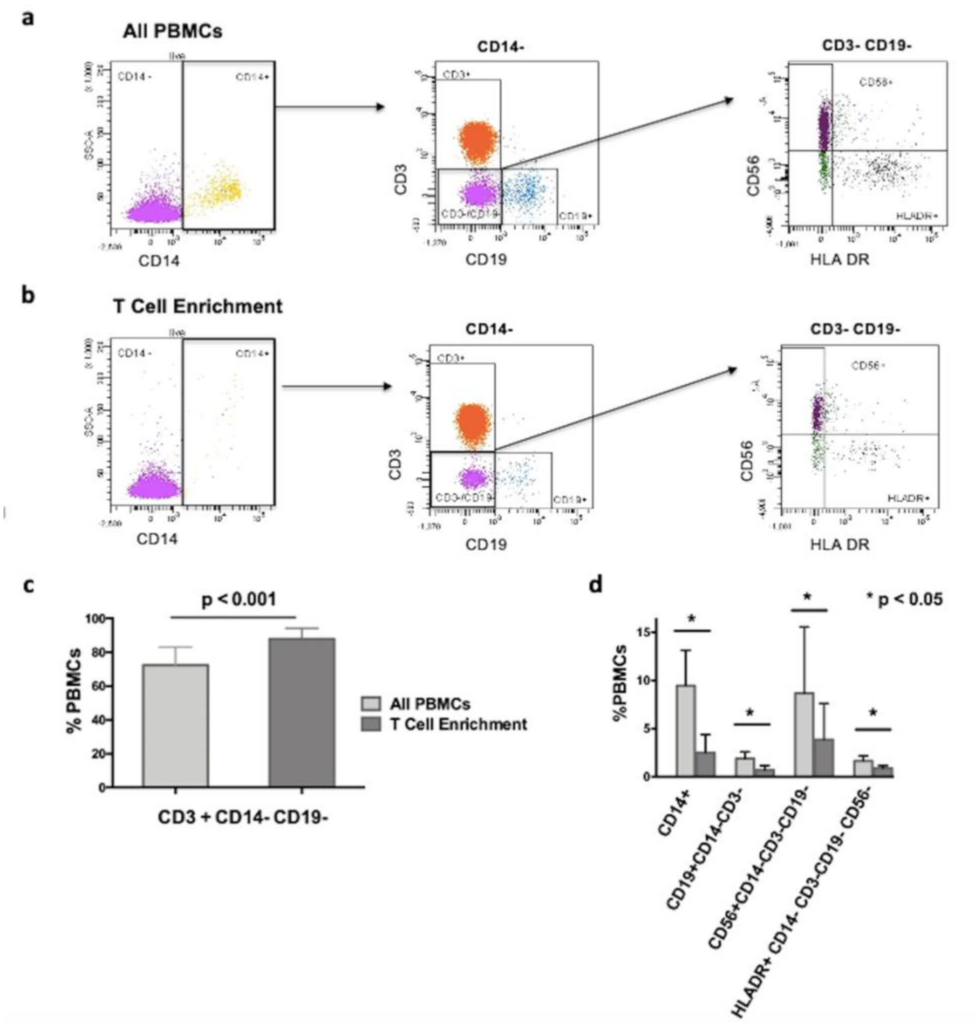

Abbreviations: PBMC: Peripheral blood mononuclear cell

Supplementary figure 2. Association between HLA allelic MM, amino acidic MM, Eplet MM load and PIRCHE-II scores

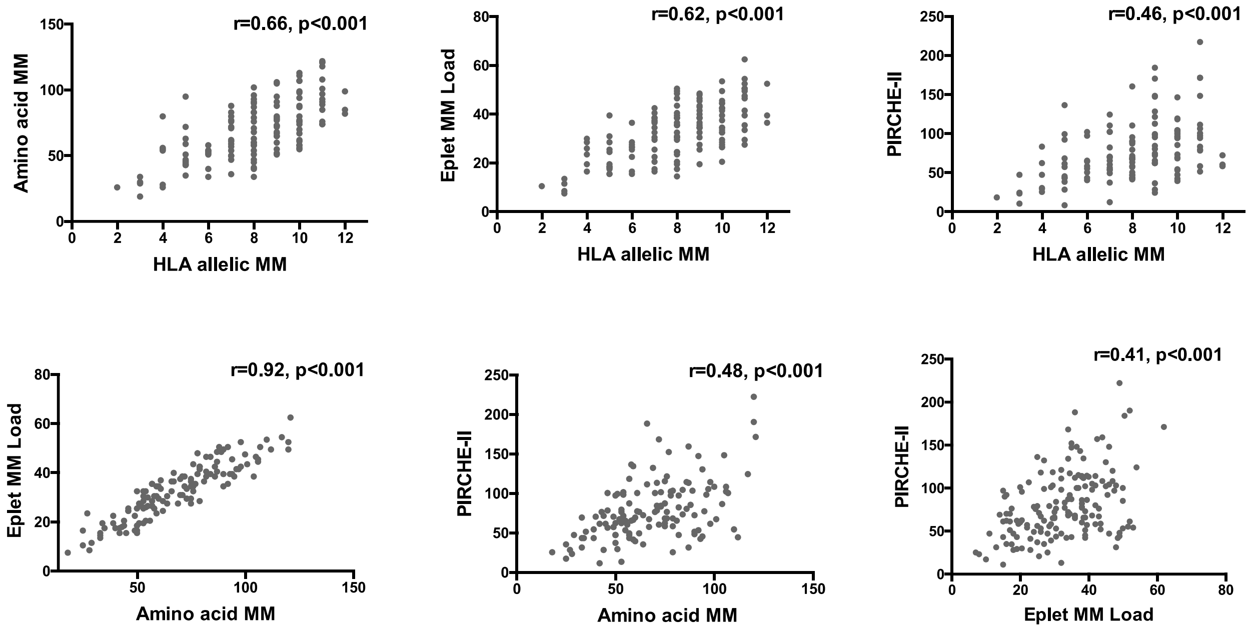

Abbreviations: MM: mismatches

Supplemental figure 3. Pretransplant DST and risk of BPAR

Kaplan–Meier curves illustrating the cumulative incidence of BPAR (**3a**), TCMR (**3b**) and ABMR (**3c**) stratified according to pretransplant DST.

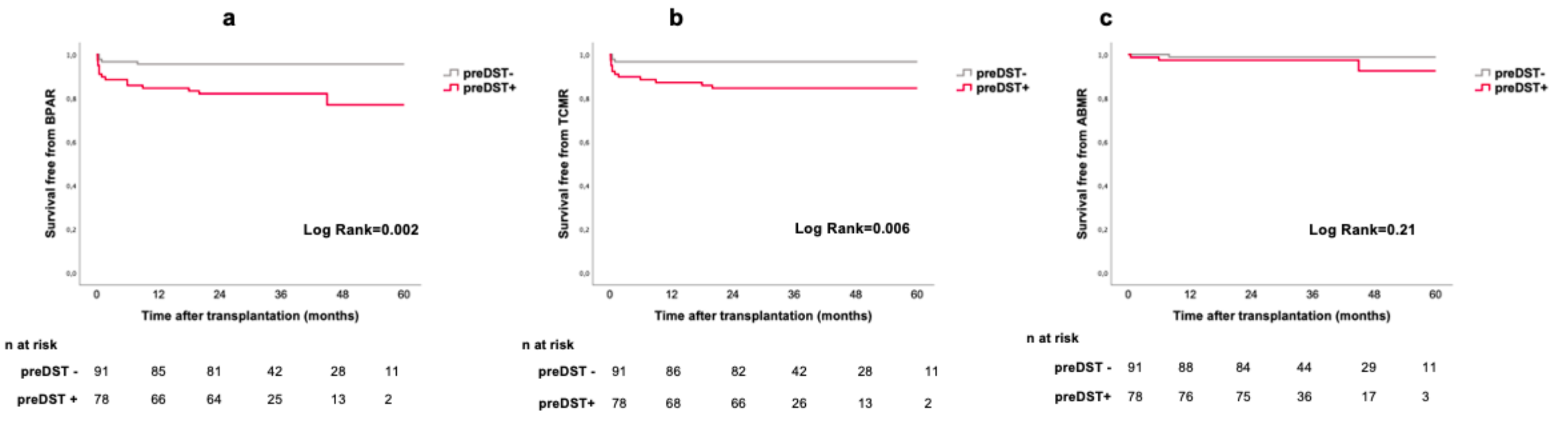

Abbreviations: BPAR: biopsy-proven acute rejection; TCMR: T-cell mediated rejection; ABMR: antibody-mediated rejection; preDST: pretransplant donor-specific T-cell alloimmune response

Supplementary figure 4. Relationship between PIRCHE-II score and dnDST for different peptide affinity thresholds.

Relation between PIRCHE-II and dnDST at different IC50 thresholds. IC50 0-50: 8.76±9.4 vs 12.6±13.9, p=0.11; IC50 0-125: 19.2±15 vs 27.6±23.6, p=0.04; IC50 250-1000: 51.07±20.5 vs 64±26.1, p=0.01 for DST- and dnDST, respectively.

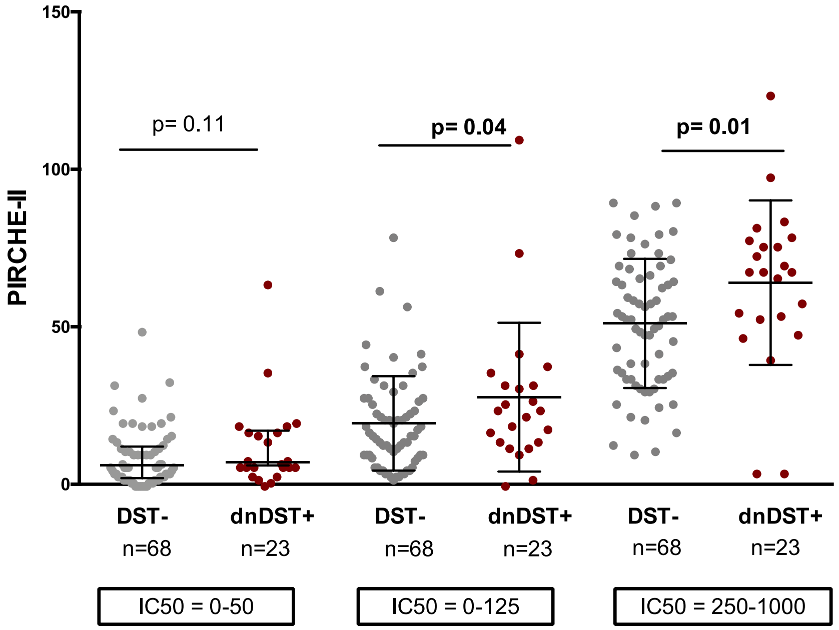

Abbreviations: DST: donor-specific; dnDST *de novo* donor-specific T-cell alloimmune response

Supplemental figure 5. Representative FACS plot gating strategy for negative control, Allo DST negative test, pretransplant DST positive test and *de novo* posttransplant DST test.

AIM+ Cells were defined as % of living cells expressing CD69 and CD137 for CD8+T cells and CD134-OX40 and CD137 for CD4+T cells, respectively. All results are expressed subtracting the percentage of AIM+ cells after stimulation with medium (negative control) from % of AIM+ cells after allogenic stimulation

5a. Negative control (medium): median 0.02 [0.012-0.076] for CD4+AIM+ and 0.45 [0.29-0.96] for CD8+AIM+.

5b. Allo DST negative test: median 0.08% [0.008-0.19] for CD4+AIM+ and 0.17% [0.05-0.42] for CD8+AIM+

5c. pre transplant DST positive test: median 0.25% [0.14-0.62] for CD4+AIM+ and 1.2% [0.65-3.55] of CD8+AIM+

5d. *de novo* posttransplant DST test: median 0.4% [0.14-0.53] for CD4+AIM+ and 0.60% [0.25-1.2] for CD8+AIM+.

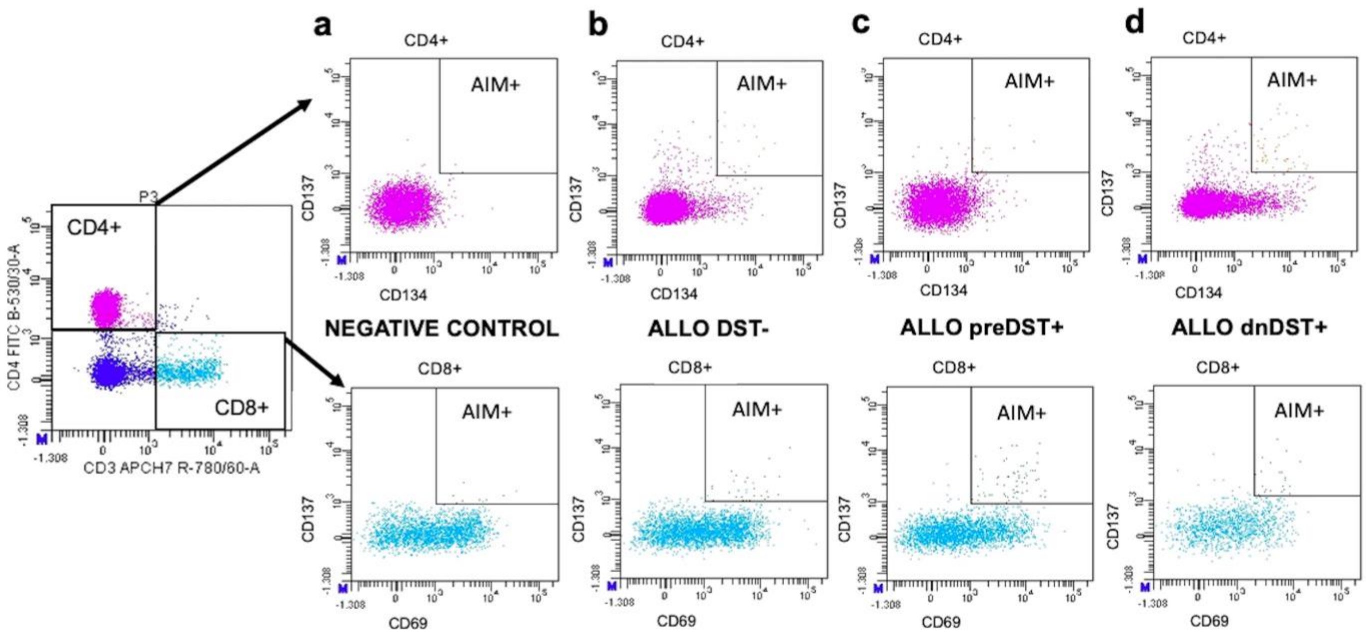

Abbreviations: AIM: T-cell receptor dependent activation-induced cell markers (AIM); DST: donor-specific T-cell alloimmune response; preDST: pretransplant donor-specific T-cell alloimmune response; dnDST *de novo* donor-specific T-cell alloimmune response

Supplementary Tables

Supplementary table 1. Donor/recipient HLA allelic, amino acidic MM, HLAMatchmaker Eplet and PIRCHE-II of all patients of the study.

Donor/Recipient HLA MM scores	All patients<br>N=169
**HLA allelic MM**	8 (7-10)
Class I (A, B, C)	4 (3-5)
Class II (DRB1, DQB1; DPB1)	4 (3-5)
**Amino acidic HLA MM / Solvent accessible**	69.5 (51-87)
A	15 (7,5-23) / 11 (5-17)
B	14 (9-18) / 6 (4-9)
C	11 (6-15) / 6 (2-9)
DRB1	11 (4-17) / 8 (3-14)
DQB1	12 (3-18) / 8 (2-13)
DPB1	4 (1-10.25) / 2 (1-7)
**Eplet MM Load / Antibody verified**	33 (24-40)
Eplet Class I (A+B+C)	15 (11-19) / 9 (6-11)
Eplet Class II	19 (11-25) / 7 (4-9)
Eplet DRB1	8 (3-12) / 3 (1-5)
Eplet DQB1	7 (2-10) / 2 (1-4)
Eplet DPB1	2 (1-5) / 1 (0-2)
**PIRCHE-II score *(originated peptides per locus)***	71 (51-102)
PIRCHE-II A	14 (6-25)
PIRCHE-II B	14 (6-25)
PIRCHE-II C	12 (8-22)
PIRCHE-II DRB1	10 (4-16)
PIRCHE-II DQB1	17 (7-26)
PIRCHE-II DPB1	3 (0-7)

Data are median (Inter-quartile Range: IQR).

Abbreviations: MM: mismatch

Supplementary table 2. Comparison of main baseline characteristics and clinical outcomes according to pretransplant DST.

Main baseline characteristics	preDST- (n= 91)	preDST+ (n=78)	P value
Recipient age (Years)	51.5±14	51.9±14	0.84
Recipient gender (Male)	60 (66)	50 (64)	0.80
Race (Caucasic)	86 (95)	72 (92)	0.56
Cause of End Stage disease			0.77
Vascular	10 (11)	10 (13)	
Diabetes	4 (4)	4 (5)	
Glomerular	28(31)	20 (26)	
Polycystic Kidney disease	15 (16)	8 (10)	
Interstitial disease	12 (13)	12 (15)	
Others/unknown	22 (24)	24 (31)	
Time on dialysis (months)	26.32±35	24.19±33	0.69
Transplant type (deceased)	59 (65)	56 (72)	0.33
Transplant number (1)	83 (91)	69 (88)	0.55
Cold ischemia time (Hours)	12.34±10	13.25±9.4	0.55
Pre-transplant anti-HLA antibodies (cPRA)			
- Class I	10 (12)	7 (9)	0.51
- Class II	22 (27)	16 (21)	0.35
**Main Clinical Outcomes**			
DGF	22 (24)	24 (31)	0.36
BPAR	4 (4)	15 (19)	**0.002**
TCMR	3	12	**0.006**
ABMR	1	3	0.24
De novo DSA	13 (14)	11 (14)	
HLA Class I	3 (23)	3 (27)	0.97
HLA Class II	11 (85)	8 (73)	
HLA Class I&II	0	1 (9)	
eGFR (ml/min/1.73m^2)			
12 months (n=160)	54.52±17.7	55.31±18.7	0.77
24 months (n=157)	53.38±17.7	52.49±19.0	0.76
36 months (n=146)	52.32±19.2	50.73±17.3	0.60
Death-censored graft loss	5 (6)	4 (5)	0.87
Patient death	7 (8)	4 (5)	0.50

Abbreviations: cPRA: calculated panel of reactive antibodies; rATG: rabbit anti thymoglobulin; CNI: Calcineurin inhibitor; DGF: delayed graft function; eGFR: estimated glomerular filtrate rate (CKD-EPI); BPAR: Biopsy-proven acute rejection; TCMR: T cell mediated rejection; ABMR: antibody-mediated rejection; DSA: donor-specific antibodies.

Supplementary table 3. DST frequencies primed by the direct and indirect pathway of antigen presentation

	Global postDST	Frequencies of DP_DST	Frequencies of IP_DST	Presence of IP	HLA allelic MM	Amino acid MM	Eplet MM Load	Global PIRCHE-II
				**preDST+**				
1	108	80	28	Yes	8	50	32	46
2	85	50	35	Yes	6	25	37	141
3	69	78	0	No	6	60	33	41
4	32	32	0	No	9	51	19	26
5	36	40	0	No	7	79	42	57
6	31	35	0	No	7	87	40	112
7	234	234	0	No	8	59	23	30
8	95	100	0	No	8	40	8	69
9	26	30	0	No	4	55	17	65
				**dnDST+**				
1	54	35	20	Yes	7	46	25	55
2	68	6	60	Yes	8	89	48	102
3	52	40	12	Yes	9	63	36	187
4	91	70	21	Yes	8	51	29	102
5	250	30	200	Yes	7	71	38	112
6	45	65	0	No	6	41	28	55
7	41,5	45	0	No	5	42	15	10
8	79,3	80	0	No	7	52	29	100
9	33	33	0	No	8	92	50	52
				**persistDST+**				
1	44	35	10	Yes	9	51	19	26
2	53	19,5	30	Yes	10	57	28	53
3	80.5	38	40	Yes	9	104	38	85
4	65	45	20	Yes	8	50	32	46
5	79	51	30	Yes	8	40	8	69
6	71	80	0	No	10	61	33	67
7	60.8	70	0	No	6	58	33	41
8	36	55	0	No	7	79	42	57
9	234	230	0	No	8	59	23	30

Abbreviations: DST: donor specific T cell alloreactivity (DP_DST: direct pathway; IP_DST indirect pathway; postDST: post-transplant DST; dnDST: *de novo* DST; persistDST: persistent DST). MM: mismatch
